# Incidence of suicidality in people with depression over a 10-year period treated by a large UK mental health service provider

**DOI:** 10.1192/bjo.2021.1054

**Published:** 2021-11-24

**Authors:** Emma R. Francis, Daniela Fonseca de Freitas, Craig Colling, Megan Pritchard, Giouliana Kadra-Scalzo, Natalia Viani, Jaya Chaturvedi, Tom R. Denee, Cicely Kerr, Mitesh Desai, Gemma Scott, Hitesh Shetty, Mathew Broadbent, David Chandran, Johnny Downs, Sumithra Velupillai, Mizanur Khondoker, Robert Stewart, Rina Dutta, Richard D. Hayes

**Affiliations:** Institute of Psychiatry, Psychology and Neuroscience, King's College London, UK; and Division of Psychology and Language Sciences, University College London, UK; Institute of Psychiatry, Psychology and Neuroscience, King's College London, UK; South London and Maudsley NHS Foundation Trust, UK; South London and Maudsley NHS Foundation Trust, UK; Institute of Psychiatry, Psychology and Neuroscience, King's College London, UK; Institute of Psychiatry, Psychology and Neuroscience, King's College London, UK; Institute of Psychiatry, Psychology and Neuroscience, King's College London, UK; Janssen-Cilag Limited, High Wycombe, UK; Janssen-Cilag Limited, High Wycombe, UK; Janssen-Cilag Limited, High Wycombe, UK; Janssen-Cilag Limited, High Wycombe, UK; South London and Maudsley NHS Foundation Trust, UK; South London and Maudsley NHS Foundation Trust, UK; Institute of Psychiatry, Psychology and Neuroscience, King's College London, UK; and South London and Maudsley NHS Foundation Trust, UK; Institute of Psychiatry, Psychology and Neuroscience, King's College London, UK; and South London and Maudsley NHS Foundation Trust, UK; Institute of Psychiatry, Psychology and Neuroscience, King's College London, UK; Norwich Medical School, University of East Anglia, Norwich, UK; Institute of Psychiatry, Psychology and Neuroscience, King's College London, UK; and South London and Maudsley NHS Foundation Trust, UK; Institute of Psychiatry, Psychology and Neuroscience, King's College London, UK; and South London and Maudsley NHS Foundation Trust, UK; Institute of Psychiatry, Psychology and Neuroscience, King's College London, UK

**Keywords:** Suicidal ideation, high risk of suicide, incidence, electronic health records, depression

## Abstract

We describe the incidence of suicidality (2007–2017) in people with depression treated by secondary mental healthcare services at South London and Maudsley NHS Trust (*n* = 26 412). We estimated yearly incidence of ‘suicidal ideation’ and ‘high risk of suicide’ from structured and free-text fields of the Clinical Record Interactive Search system. The incidence of suicidal ideation increased from 0.6 (2007) to 1 cases (2017) per 1000 population. The incidence of high risk of suicide, based on risk forms, varied between 0.06 and 0.50 cases per 1000 adult population (2008–2017). Electronic health records provide the opportunity to examine suicidality on a large scale, but the impact of service-related changes in the use of structured risk assessment should be considered.

Monitoring changes in suicidality in depression over time is important for understanding the burden this places on individuals, healthcare services and society. In England and Wales, the most recent Office for National Statistics report of registered deaths by suicide observed that there were 11.0 deaths per 100 000 population (age-standardised rate).^1^ Although the majority of people diagnosed with depression do not die by suicide, they do have an increased risk compared with the rest of the population.^2^ To our knowledge, there are no studies that have described the incidence of high risk of suicide and of suicidal ideation among people with depression treated in UK secondary mental healthcare. The aims of this report were to identify the incidence of (a) high risk of suicide and (b) suicidal ideation among people diagnosed with depressive disorder treated in secondary mental healthcare.

## Method

Patient data were extracted from the Clinical Record Interactive Search (CRIS) database at the South London and Maudsley NHS Foundation Trust (SLaM). CRIS permits search and retrieval of information in the de-identified electronic health records (EHRs) for research purposes.^3^ SLaM is a large provider of comprehensive mental health services for approximately 1.3 million people situated in four London boroughs: Lewisham, Southwark, Lambeth and Croydon.^4^ We assembled the sample for analysis using a combination of routinely collected data from structured fields and natural language processing (NLP) algorithms that captured data from free-text fields.

Inclusion criteria:

- primary diagnosis of a depressive episode or recurrent depressive disorder (F32–F33) according to ICD-10;^5^
- aged 18 years or older at the time of diagnosis;
- at least one general practitioner or home address within the four catchment London boroughs at any time point during the observation period from 1 January 2007 to 31 December 2017.

### Identifying patients at high risk of suicide

The term ‘high risk’ in this report represents a global assessment by a clinician about the risk of suicide as indicated in the EHR risk assessments that are routinely completed trust-wide throughout the observation period. These comprised the ‘full risk assessment form’ (administered from 2008) or the ‘risk assessment tool’ (administered from 2016). To be consistent, we selected items referring to current plans to end life across both forms. On the full risk assessment form the item ‘Has patient made plan to end life?’ had to be assigned ‘Yes’. On the risk assessment tool the items ‘Self harm/suicide’ and ‘Expressed plans to end life’ were assigned ‘Yes’ and ‘Current: Yes’. Patients must have met criteria for high risk of suicide on either of the forms.

### Identifying patients with suicidal ideation

The term ‘suicidal ideation’ used in this report refers to a patient thinking or planning to take their own life as recorded by a clinician in the EHR. Suicidal ideation was identified using an NLP algorithm that applies basic rules to free-text fields. These rules identify sentences containing the common terms used in research and clinical settings: ‘suicid*’ and ‘ideat*’ (the asterisk denotes a wild card for any combination of letters following).^6^ Unlike a keyword search, the NLP algorithm takes into account contextual information around the term of interest. This allows automated coding of entities from free text^7^ and enables them to be distinguished from negation statements and irrelevant text entities to identify patients (precision (positive predictive value): 92%; recall (sensitivity): 88%).^6^ Examples of free-text fields searched by the algorithm are events (day-to-day notes) and correspondence between patient and clinician or between clinicians about the patient. We identified patients using NLP as there is evidence to suggest that using free-text fields might increase the number of patients in our sample, as clinicians may not record the presence of suicidal ideation using ICD codes (structured fields).^8^

It is important to note that complex terms or phrases used by the clinician, such as ‘planning to end life’, will not have been classified as a positive statement of suicidal ideation by this algorithm as these terms do not feature in the basic rules applied. A recorded suicide attempt was also not classified as a positive statement of suicidal ideation. Further details as to what is captured by the algorithm can be found in Fernandes et al (2018).^6^

### Ethics statement

The authors assert that all procedures contributing to this work comply with the ethical standards of the relevant national and institutional committees on human experimentation and with the Helsinki Declaration of 1975, as revised in 2008. The use of CRIS as a database for secondary analysis was approved by Oxford Research Ethics Committee C (18/SC/0372). The data is used in an anonymised and data-secure format under strict governance procudures. CRIS data is available to researchers with appropriate credentials (provided by the South London and Maudsley NHS Trust). Projects are approved by a CRIS oversight committee, a body set up by and reporting to the South London and Maudsley Caldicott Guardian. Individual patient consent was not required for the use of the de-identified CRIS data.

### Data analysis

Analyses were conducted using STATA for Windows, release 5.^9^ When calculating incidence of high risk of suicide and suicidal ideation (separately), cases contributed to the numerator for the year of the first entry of each of these forms of suicidality and could not contribute to the numerator or denominator in subsequent years. This was because they were no longer considered part of the at-risk population for the condition being studied, as we were only interested in the first report of suicidality in the observation period.^10^ To calculate incidence, the annual adult (≥18 years) population mid-year estimates (2007–2017) for Lewisham, Southwark, Lambeth and Croydon, drawn from the Office for National Statistics database, were used as the denominator.^11^ The incidence of high risk of suicide in our sample is reported from 2008, which is the time point at which risk forms became electronically accessible. To gain insight into possible changes in the use of the risk forms between 2008 and 2017, we calculated the number of former (‘full risk assessment form’ from 2008), current (‘risk assessment tool’ from 2016) and total risk assessments (both risk assessment forms). The incidence of suicidal ideation was calculated between the years 2007 and 2017.

## Results

There were 26 412 patients diagnosed with depression (F32x–F33) treated in secondary care during 2007–2017. Table 1 details the sociodemographic, socioeconomic and clinical characteristics of these patients. In the high risk of suicide group (mean age: 45.4 years) there was a similar proportion of men and women (49% and 51%); however, the suicidal ideation group (mean age: 44.2 years) had a lower proportion of men than women (41% and 59%). Both groups had a higher distribution of patients identified as White (high risk of suicide: 67.8%; suicidal ideation: 63.2%). The majority of patients in both groups lived in the socioeconomically most deprived areas of England. Neurotic, stress-related and somatoform disorders (F40–F48) were the most common psychiatric comorbidities in both groups.

**Table 1:** Descriptive data for patients receiving secondary care treatment for depression by group (‘at high-risk of suicide’ and ‘with suicidal ideation’)

Variables	High risk of suicide (*n* = 1328)	Suicidal ideation (*n* = 9547)
**Sociodemographic and socioeconomic characteristics**		
Gender, *n* (%, 95% CI)		
Male	652 (49.1, 46.3–51.8)	3912 (41.0, 40.0–42.0)
Female	675 (50.8, 48.1–53.6)	5633 (59.0, 58.0–60.0)
Other	<10	<10
Not specified	<10	<10
Not known	<10	<10
Age, years		
Mean (s.d); min–max	45.4 (17.1); 18.0–97.3	44.2 (17.4); 18.0–98.4
Median (IQR)	44.4 (31.5−55.0)	41.8 (30.4−53.7)
Age group, years: *n* (%, 95% CI)		
18–29	294 (22.1, 19.9–24.5)	2315 (24.3, 23.4–25.1)
30–39	254 (19.1, 17.0–21.3)	2062 (21.6, 20.8–22.4)
40–49	320 (24.1, 21.8–26.5)	2128 (22.3, 21.5–23.1)
50–59	213 (16.0, 14.1–18.1)	1381 (14.5, 13.8–15.2)
60–69	109 (8.2, 6.8–9.8)	648 (6.8, 6.3–7.3)
70–79	87 (6.6, 5.3–8.0)	557 (5.8, 5.3–6.3)
80–89	40 (3.0, 2.2–4.1)	382 (4.0, 3.6–4.4)
90–99	11 (0.8, 0.4–1.4)	74 (0.8, 0.6–1.0)
Ethnic group: *n* (%, 95% CI)		
White	880 (67.8, 65.2–70.4)	5828 (63.2, 62.2–64.2)
Black/African/Caribbean/Black British	208 (16.0, 14.1–18.1)	1752 (19.0, 18.2–19.8)
Asian/Asian British	81 (6.2, 5.0−7.7)	525 (5.7, 5.2–6.2)
Mixed/multiple ethnic groups	25 (1.9, 1.3–2.8)	208 (2.3, 2.0–2.6)
Other ethnic group	103 (7.9, 6.5–9.5)	911 (10.0, 9.3–10.5)
Not stated or missing	31 (2.3)	323 (3.4)
Neighbourhood deprivation, based on the English Index of Multiple Deprivation deciles: *n* (%, 95% CI)		
1–2: 20% most deprived	462 (36.2, 33.6–38.9)	3487 (37.5, 36.5–38.5)
3–4: 20% second most deprived	561 (44.0, 41.3–46.8)	4082 (43.9, 42.9–44.9)
5–6: 20% middle deprivation	163 (12.8, 11.0–14.7)	1216 (13.1, 12.4–13.8)
7–8: 20% second least deprived	64 (5.0, 3.9–6.4)	375 (4.0, 3.6–4.5)
9–10: 20% least deprived	25 (2.0, 1.3–2.9)	137 (1.5, 1.2–1.7)
Missing	53 (4.0)	250 (2.6)
Homeless (ever) with no address at index: *n* (%, 95% CI)		
	41 (3.1, 2.2–4.1)	175 (1.8, 1.5–2.1)
**Clinical factors**		
Earliest ICD-10 diagnosis that met the study criteria: *n* (%, 95% CI)		
Depressive episode (F32x)	1034 (77.9, 75.5–80.1)	7456 (78.1, 77.2–78.9)
Recurrent depressive disorder (F33x)	294 (22.1, 19.9–24.5)	2091 (21.9, 21.1–22.7)
Time from depressive disorder diagnosis to identification of high-risk of suicide or suicidal ideation, days		
Mean (s.d.)	774.4 (992.3)	382.4 (686.2)
Median (IQR)	330.5 (15.0–1101.0)	43.0 (1.0–429.0)
Range	0−3971	0–3796
Psychiatric comorbidity: *n* (%, 95% CI)		
Personality disorder (F60)	270 (20.3, 18.2–22.6)	792 (8.3, 7.8–8.9)
Substance-use disorder (F10–F19)	246 (18.2, 16.2–20.4)	1113 (11.7, 11.0–12.3)
Neurotic, stress-related and somatoform disorders (F40–F48)	386 (29.1, 26.6–31.6)	2111 (22.1, 21.3–23.0)
Bipolar disorder (F31*x*)	68 (5.1, 4.0–6.4)	292 (3.1, 2.7–3.4)
Schizophrenia (F20–F29)	113 (8.5, 7.1–10.1)	552 (5.8, 5.3–6.3)
Organic disorder (F0*x*)	59 (4.4, 3.4–5.7)	428 (4.5, 4.1–4.9)
**Service use**		
First week		
Patients who had at least one in-patient stay, *n* (%, 95% CI)	567 (42.7, 40.0–45.4)	1697 (17.8, 17.0–18.6)
In-patient days, *n*: mean (s.d.), median (IQR)	6.2 (2.5), 8 (4–8)	6.1 (2.5), 8 (4–8)
First month		
Patients who had at least one in-patient stay, *n* (%, 95% CI)	598 (45.0, 42.3–47.8)	1845 (19.3, 18.5–20.1)
In-patient days, *n*: mean (s.d.), median (IQR)	16.3 (11.7), 15 (5–30)	16.7 (11.6), 15 (5–30)
Six months		
Patients who had at least one in-patient stay, *n* (%, 95% CI)	641 (48.3, 45.5–51.0)	2143 (22.5, 21.6–23.3)
In-patient days, *n*: mean (s.d.), median (IQR)	34.3 (43.2), 18 (6–43)	37.3 (45.1), 19 (6–50)
Twelve months		
Patients who had at least one in-patient stay, *n* (%, 95% CI)	657 (49.5, 46.7–52.2)	2303 (24.1, 23.3–25.0)
In-patient days, *n*: mean (s.d.), median (IQR)	42.3 (63.3), 20 (6–47)	46.0 (65.3), 21 (6–56)
Patients detained under MHA section in the 12 months following the identification of suicidality: *n* (%, 95% CI)		
	206 (15.5, 13.6–17.6)	700 (7.3, 6.8–7.9)

Of the total sample, 1328 (5.03%) were identified as being at high risk of suicide over the observation period. Although the new risk assessment tool was introduced in 2016, the previous risk assessment form continued to be used beyond 2016, albeit at low levels (219 administered in 2017). The incidence of high risk of suicide, based on these forms, varied between 0.06 and 0.50 cases per 1000 adult population, also highest in 2016 and 2017 (Supplementary Figs 1 and 2, available at https://doi.org/10.1192/bjo.2021.1054). The incidence of high risk of suicide closely followed the number of risk forms administered.

Of the total sample, 9547 (36.15%) were identified as having suicidal ideation between 2007 and 2017. Within the 11-year observation period, the yearly incidence of depression with suicidal ideation varied between 0.61 and 1.12 cases per 1000 adult population and was highest in 2016 and 2017 (Supplementary Fig. 3).

## Discussion

To our knowledge, this is the first study to investigate the incidence of suicidality in depression in adults engaged with UK secondary care services. We found a rising trend in suicidal ideation between the years 2007 and 2017 which may indicate an increase in suicidality in this referred population. Alternatively, this may be partially explained by the changes in the nature or circumstances of the catchment population. After 2015, there was an exponential increase in the incidence of high risk of suicide which may be due to trust-wide clinical practice changes such as the observed increase in the use of risk assessment forms.

The most recent National Confidential Inquiry into Suicide and Safety in Mental Health found that 20% of people who died by suicide had had contact with mental healthcare services within the prior 12 months.^12^ However, a systematic review of the literature observed that measuring suicide ideation alone is not sensitive enough to predict suicide, finding that 60% of people who died by suicide had not expressed suicidal ideation.^13^

This investigation has several strengths. CRIS features a wealth of patient-level longitudinal data that include demographic and clinical characteristics that may be used to identify patients at risk. As SLaM is close to being a monopoly mental healthcare provider for the four London boroughs it serves, our sample is likely to be broadly generalisable to patients treated in secondary care in similarly ethnically diverse and deprived areas in the UK.^4^

However, there will be people who experience suicidality and do not receive secondary care assessment, because their depression remains undiagnosed or is managed in primary care alone. Further to this, owing to how we identified patients we are unable to determine whether the severity of illness increased over the years or whether more people were diagnosed with depression over the years. In addition, it is important to acknowledge that, as the EHR became available trust-wide from 2008, there may have been a steep increase in reported events from 2008–2009 owing to changes in the use of the risk forms during this transition period. However, regarding the incidence of suicidal ideation we have no reason to believe this was affected by implementation of the electronic health records. We expect that clinicians would describe this condition in a similar way whether this be in an electronic or paper-based record. We observe more of a steady increase in suicidal ideation over time rather than a sharp increase in the first few years.

Our findings show that clinical reporting can have a notable impact on incidence estimates. Future research should investigate the consistency of these trends across other sites using routinely collected data and determine whether there is an association between high risk of suicide and suicidal ideation groups by obtaining datasets that feature recurrent events in the same year. Investigating the associations between the variables described and outcomes discussed in this short report may provide new insights.

## Data Availability

A dedicated CRIS Security Model has been developed to ensure that the ethical and legal rights of patients are protected. Access to CRIS data requires approval by the CRIS Oversight Committee.

## Supplementary material

For supplementary material accompanying this paper visit http://dx.doi.org/10.1192/bjo.2021.1054. click here to view supplementary material
