# Supplementary material for: Incidence of suicidality in people with depression over a 10-year period treated by a large UK mental health service provider
Source: BJPsych Open. 2021 Nov 24;7(6):e223. doi: 10.1192/bjo.2021.1054 (PMC8693911; doi:10.1192/bjo.2021.1054)
Supplement: Supplementary file 1 [file S2056472421010541sup001.docx]

**SUPPLEMENTARY MATERIAL (FIGURES 1-3)**

*The incidence of high-risk of suicide with secondary-care treated depression*

**Figure 1:** Incidence calculated as the proportion of secondary-care treated depression cases newly recorded with high-risk of suicide in the four London boroughs (Lewisham, Southwark, Lambeth, Croydon) (2008-2017) each year. The incidence is shown by the black solid line. Upper and lower 95% confidence intervals are shown by the dark grey dotted lines above and below the incidence line.

*The total number of risk assessment forms administered in South London and Maudsley NHS Trust*

**Figure 2:** The total number of risk assessment forms was calculated from the full risk assessment forms (2008-2016) shown by the black line on the graph, and the risk screen tool administered Trust-wide from 2016 which is represented by the dark grey line on the graph.

*The incidence of secondary-care treated depression with recorded suicidal ideation*

**Figure 3:** Incidence calculated as the proportion of secondary-care treated depression with newly recorded suicidal ideation in the four boroughs (Lewisham, Southwark, Lambeth, Croydon) (2007-2017) each year. The incidence is shown by the black solid line. Upper and lower 95% confidence intervals are shown by the dark grey dotted lines above and below the incidence.
